# Stereoselective Processes Based on σ-Hole Interactions

**DOI:** 10.3390/molecules27144625

**Published:** 2022-07-20

**Authors:** Paola Peluso, Victor Mamane

**Affiliations:** 1 Istituto di Chimica Biomolecolare ICB, CNR, Sede Secondaria di Sassari, Traversa La Crucca 3, Regione Baldinca, Li Punti, 07100 Sassari, Italy; 2 Institut de Chimie de Strasbourg, UMR CNRS 7177, Equipe LASYROC, 1 Rue Blaise Pascal, 67008 Strasbourg, France

**Keywords:** chalcogen bond, enantioselectivity, enantioseparation, halogen bond, σ-hole, noncovalent interaction, organocatalysis, recognition

## Abstract

The σ-hole interaction represents a noncovalent interaction between atoms with σ-hole(s) on their surface (such as halogens and chalcogens) and negative sites. Over the last decade, significant developments have emerged in applications where the σ-hole interaction was demonstrated to play a key role in the control over chirality. The aim of this review is to give a comprehensive overview of the current advancements in the use of σ-hole interactions in stereoselective processes, such as formation of chiral supramolecular assemblies, separation of enantiomers, enantioselective complexation and asymmetric catalysis.

## 1. Introduction

σ-hole interactions encompass all noncovalent interactions between atoms with σ-hole(s) on their surface (σ-hole donors) and negative sites (σ-hole acceptors: anions, lone electron pairs or π electrons). The term “σ-hole” was introduced by Politzer et al. in order to define the region of positive electrostatic potential mapped on a density isosurface (*V*_S_) observed in a series of covalently bonded atoms, such as halogens (Cl, Br, I) [1,2], chalcogens (S, Se, Te) [3] and pnictogens (N, P, As, Sb, Bi) [4]. The number of σ-holes in each atom is directly related to its covalency; thus, halogens possess one σ-hole, whereas chalcogens possess two σ-holes and pnictogens three. For these interactions, more specific names in relation to the nature of the atom in the interaction through its σ-hole(s) are generally employed in the literature, i.e., halogen bond (acronym XB) [5,6], chalcogen bond (ChB) [7] and pnictogen bond (PnB) [8,9]. In recent years, the list of σ-hole interactions was enriched, since many other atoms were shown to have σ-holes. The current list contains tetrel bond (TeB for C, Si, Ge, Sn, Pb) [10], aerogen or noble gas bond (NgB for Ar, Kr, Xe) [11], regium or coinage-metal bond (CiB for Cu, Ag, Au, Pt) [12,13], spodium bond (SpB for Zn, Cd, Hg) [14], osme bond (OmB for Fe, Ru, Os) [15] and wolfium bond (WfB) [16].

**Figure 1:**
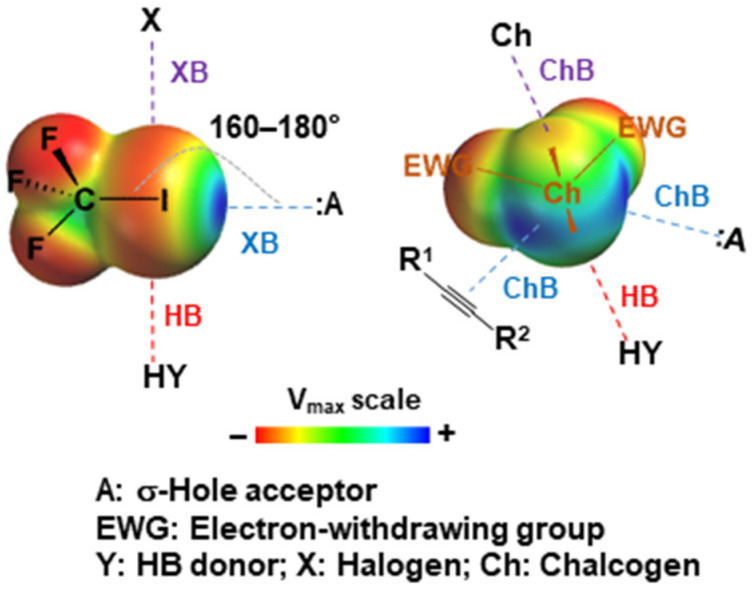
Halogen bond (XB) and chalcogen bond (ChB) as representative members of σ-hole interactions.

More precisely, σ-holes result from the anisotropy of charge distribution in covalently bonded atoms appearing in *V*_S_. representations on electron isodensity surfaces as depleted areas (often positive) of *V* on the outer sides opposite to the covalent bonds. Consequently, the σ-hole acceptors approach the σ-hole sites in a very directional manner, almost linearly to the bond involving the σ-hole donor (angles generally between 160 and 180°). It is worth noting that the remaining regions of electron density, especially those containing lone-pair electrons, appear as negatively charged areas able to interact with electron-deficient atoms, such as hydrogens, to form hydrogen bonds (HB) [17], halogens through XB (or type II halogen···halogen interactions [18]) and chalcogens through ChB (or chalcogen···chalcogen interactions [19]) (Figure 1).

While the name “σ-hole interaction” would suggest that its interaction energy is entirely dominated by electrostatics, it is important to keep in mind that it contains other components, such as charge transfer, polarization and dispersion [20].

Applications based on σ-hole interactions, especially XB and ChB, have grown rapidly over the last two decades, and important developments have resulted in crystal engineering, in biology, in supramolecular chemistry and in catalysis [6,21,22,23,24,25,26,27,28,29,30,31]. Although early studies reported the use of σ-hole interactions for inducing stereoselective processes in the solid state, their involvement in solution is much more recent. This emerging field is expected to strongly impact chirality-based applications in the next decades. The aim of this review is therefore to provide the current advancements of the field in different applications, comprising those based on chiral supramolecular assemblies (Section 2), enantioseparation (Section 3), enantioselective complexation (Section 4) and asymmetric catalysis (Section 5). Part of the latter application was recently covered in a review [32], and only the very recent examples will be examined herein.

## 2. Chiral Supramolecular Assemblies

Supramolecular chirality, defined as chirality at the supramolecular level, has found many applications in material science [33]. Supramolecular assemblies are formed through noncovalent interactions of one or several components. In these systems, chirality may arise from the specific association of either achiral or chiral components through different processes [34].

### 2.1. Spontaneous Resolution

**Figure 2:**
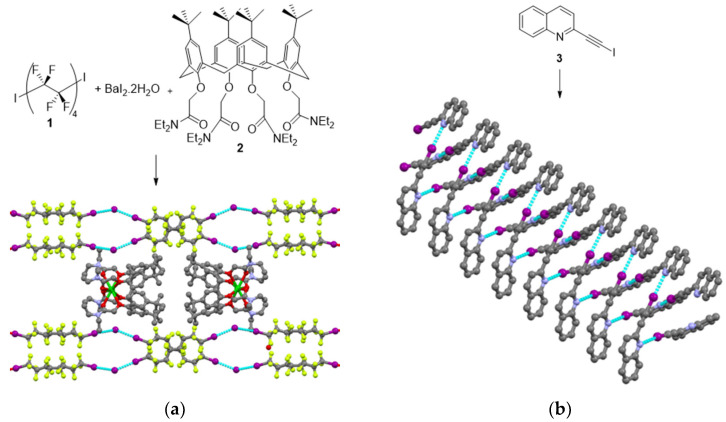
Representative examples of spontaneous resolution (XB is represented by blue dash lines): (**a**) Crystal structure of the chiral double helical structure formed by mixing **1**, **2** and BaI_2_^.^2H_2_O; (**b**) Crystal structure of the right-handed helix obtained by self-assembly of **3** (Reprinted/adapted with permission from Ref [37]. Copyright 2009, Wiley and from Ref [39]. Copyright 2018, Wiley).

Spontaneous resolution describes the process by which the segregation of enantiomers occurs upon crystallization, thus leading to conglomerates with the formation of a 1:1 mixture of homochiral crystals [35]. The first case of spontaneous resolution induced by XB was reported by the group of Metrangolo and Resnati in 2005 from the co-crystallization of 1,8-diiodoperfluorooctane **1** and *N,N,N′,N′*-tetramethyl-*p*-phenylenediamine [36]. A chiral supramolecular organization resulted from strong intermolecular XB between iodine and nitrogen. Following this work, the same group noticed that by mixing compound **1** and BaI_2_^.^2H_2_O in the presence of calixarene **2**, a chiral double helical structure could be obtained. Calixarene **2** was used for sequestration of Ba^+^, allowing I^−^ to strongly interact through XB with the diiodo derivative **1** [37] (Figure 2a). Later, Goldberg et al. showed that XB-driven spontaneous resolution could occur by self-assembly of porphyrins bearing three iodophenyl and one pyridine groups. In addition to the expected N···I contacts, other XBs implying iodine, such as I···π and I···I, were observed in the crystal [38]. More recently, Mak et al. described the spontaneous resolution of pairs of intertwined supramolecular right-handed and left-handed double helices by self-assembly of achiral 2-(iodoethynyl)quinoline **3** [39]. The chiral helical organization was mainly promoted by I···N XBs between iodoethynyl and quinoline functionalities (Figure 2b).

### 2.2. Chiral Amplification

The introduction of halogen atoms in organic building units can regulate supramolecular chirality and the corresponding chiroptical responses [40]. In XB-based chiral self-assembly systems, the control over supramolecular chirality was realized by chiral amplification with important consequences regarding their chiroptical applications.

**Figure 3:**
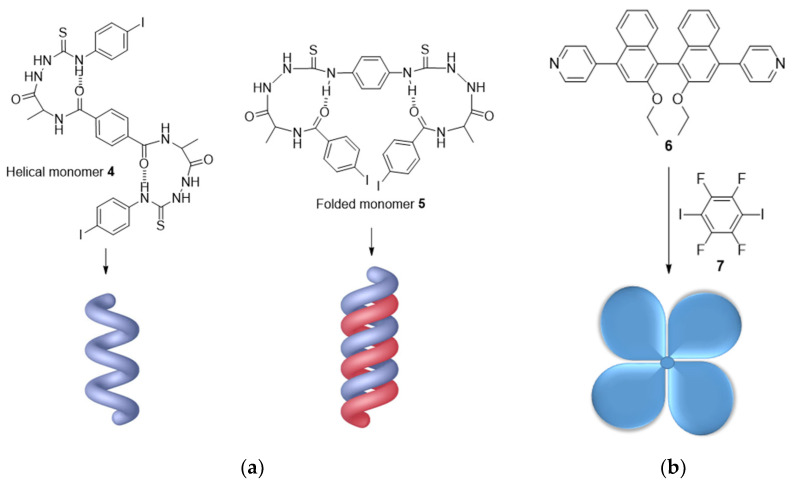
Representative examples of chiral amplification: (**a**) Formation of single or double helices through self-organization of **4** and **5**, respectively; (**b**) Formation of a chiral blade with increased chiroptical properties by combining **6** and **7**.

Chiral amplification is a phenomenon occurring during the formation of chiral supramolecular systems by strong cooperative interactions between monomers [41]. Jiang et al. applied this concept to the formation of supramolecular single and double helices through XBs [42,43]. A single-stranded helix was obtained in the solution phase by using helical amidothioureas **4** functionalized by iodophenyl groups able to interact through I^…^π XBs [42]. Structural modification of the amidothiourea allowed the folding of monomer **5**, which could now interact through I···S XBs to produce a double-stranded helix [43] (Figure 3a). By using pyrene-conjugated halogenated phenyl-alanine derivatives, XB was also shown to invert the chirality of the self-assembled helical structures [44]. While the fluorinated molecule produced a *M*-helical structure, the other compounds bearing Cl, Br and I, and therefore able to interact through XB, induced the formation of *P*-handed helices. An interesting change from helical to propeller chirality, mediated by XB, was also reported [45]. The phenomenon of chiral amplification may also result in the production of polymers with amplified chiroptical properties. This was nicely demonstrated by Jin et al. through the formation of XB-based self-assembled chiral emitters with amplified circularly polarized luminescence (CPL) [46]. While monomers **6** and **7** separately showed very weak chiroptical responses, the self-assembled chiral blades exhibited highly amplified circular dichroism (CD) and CPL signals (Figure 3b).

## 3. Enantioseparation

Although the possibility that XBs and ChBs interactions may contribute to the enantiomer distinction emerged from some studies dating back to the end of the 20th century, comprehensive studies in this field have only been reported in the last decade [47,48].

### 3.1. Enantioseparations Based on XB

Nowadays, it is known that halogen substituents can contribute to molecular recognition by playing multiple roles as nucleophiles, electrophiles, electron-withdrawing groups, hydrophobic and repulsive sites [47]. In the early 1990s, the main recognized functions of halogens in enantioseparation science for the resolution of the racemate of a chiral compound in its pure or enriched enantiomers were related to their high electronegativity and relevant electron-withdrawing properties increasing in the order I < Br < Cl < F. Hydrophobicity and polarizability were the other features recognized in halogens, which increased following the opposite order F < Cl < Br < I.

#### 3.1.1. Initial Observations on the Multiple Role of Halogens in Enantioseparation

The first observations on an “unusual” function of halogens in enantioseparation science date back to the 1990s. In those years, Caude et al. observed that chiral compounds, containing benzenoid rings acting as π-basic sites, presented reduced retention and separation factors during their enantioseparation by chiral stationary phases (CSPs) containing π-acidic sites when halogens were introduced as substituents of the π-basic sites [49].

In 1996, Pirkle et al. highlighted an opposite unusual halogen effect on the high-performance liquid chromatography (HPLC) enantioseparation of some halogenated chiral compounds by using the brush-type Whelk-O1 as the chiral column (Table 1) [50,51]. Indeed, the increase in retention and separation factors observed for the amide derivatives of l-phenyl-l-ethylamines **8** and **9** and for diethyl 1-(*N*-3,5-dimethylphenyl)amino-l-phenylmethanephosphonates **10**, featuring halogen substituents in the *meta* or *para* positions of the benzenoid ring, relative to their nonhalogenated analogs, was considered unexpected and inexplicable on the basis of the previous observations on this kind of enantioseparations [50]. The phenomenon was described by the authors as follows: “*Unexpectedly, para and meta halogen substituents increase both retention and enantioselectivity when nonaqueous organic mobile phases are used. The more polarizable the halogen, the greater the effect*” [50].

Next, the same group observed a similar behavior in the enantioseparation of 5-methyl-5-phenylhydantoins **11**, highlighting that “*contrary to one’s intuition, the electronegative halogen substituents increase enantioselectivity, the effects being greater for the more polarizable halogens […] an understanding of the principle by which halogen substituents give rise to enhanced levels of enantioselectivity can profitably be used in the design of chiral selectors and chiral catalysts*” [51]. On the one hand, a description of what we define today as the XB could be read in those words. On the other hand, Pirkle never related explicitly the halogen-dependent effects on enantioseparation to the XB, likely due to the fact that for a long time, the distribution of the electron density of halogens was considered isotropic and, consequently, F, Cl, Br and I, as substituents, were merely considered as Lewis bases in enantioseparation science [47]. Later, the identification of the anisotropic distribution of the electron density on halogens allowed for recognizing their properties as electrophiles (Lewis acid).

#### 3.1.2. Enantioseparations Based on Diastereomeric Salt Formation

**Figure 4:**
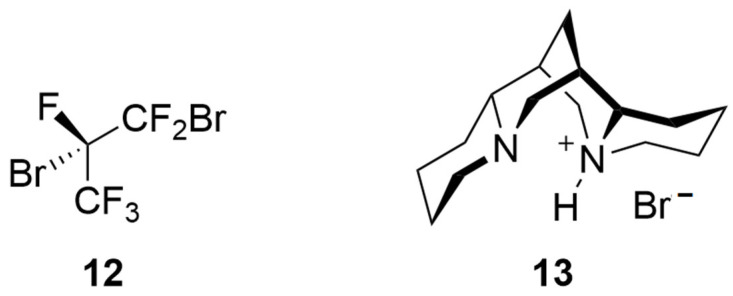
Structures of 1,2-dibromohexafluoropropane **12** and (–)-sparteine hydrobromide **13**.

The first XB-based enantiorecognition process was reported in 1999 by Resnati et al., and racemic 1,2-dibromohexafluoropropane **12** was resolved by using the Lewis base (–)-sparteine hydrobromide **13** as the chiral inductor (Figure 4) [52]. The resolution process originated from the highly specific inclusion of only the (*S*)-enantiomer of **12** in the helical-arranged chiral crystal based on the formation of a C-Br···Br^−^ interaction as XB between the C-bound Br atoms of **12** and the Br^−^ ions of **13**. The C-Br···Br^−^ distances were close to 3.3 Å, thus approximately 20% shorter than the sum of the van der Waals radii of the two bromine atoms.

Later, the same authors found that the attractive intermolecular interaction between iodine or bromine atoms of perfluorocarbon halides and nucleophilic sites of hydrocarbons can drive their self-assembly through molecular recognition [53]. On this basis, racemic perfluorocarbons were enantioseparated through an XB-driven recognition mechanism.

**Figure 5:**
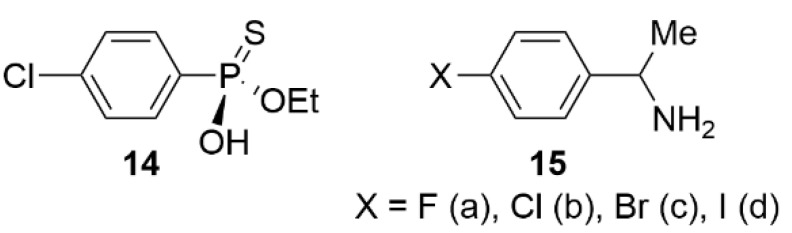
Structures of the chiral selector **14** and 1-(4-halophenyl)ethylamines (halo = F, Cl, Br, I) **15**.

In 2010, Saigo et al. synthesized and used the *O*-ethyl 4-chlorophenylphosphonothioic acid **14** as a chiral selector for the enantioseparation of racemic 1-(4-halophenyl)ethylamines (halo = F, Cl, Br, I) **15** through diastereomeric salt formation (Figure 5) [54]. The chiral selector **14** showed an excellent enantioselective recognition ability for the fluorinated and iodinated amines **15a** and **15d** with the switch of the absolute configuration of the enantio-enriched isomers in the deposited salts from *R* for the amine **15a** to *S* for the amine **15d**. X-ray crystallographic analyses of the four pairs of diastereomeric salts showed that XBs in the salt crystals were shown to play a very important role in the switch, no XB being observed in the crystal containing fluorinated **15a**.

#### 3.1.3. HPLC Enantioseparations

**Figure 6:**
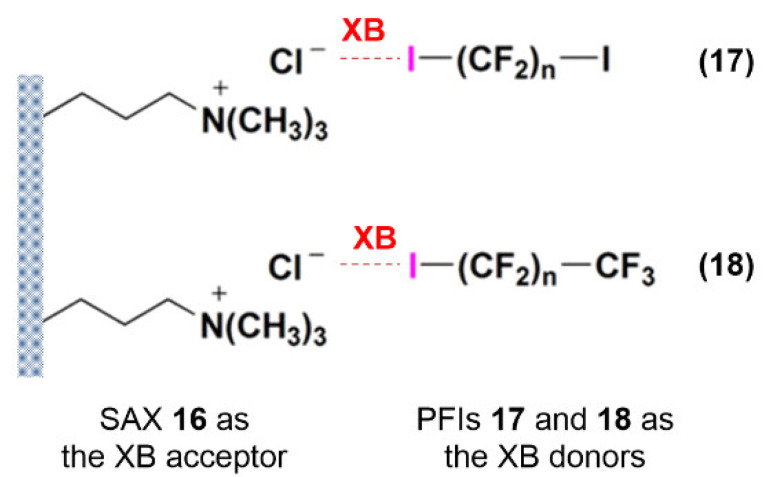
Model for the interaction of iodine (PFIs **17** and **18**) and Cl^−^ (SAX sorbent **16**).

Although not directly related to enantioseparation science, in 2012, Jin et al. described the unprecedented utilization of XB in the solid phase extraction of perfluorinated iodoalkanes (PFIs) from *n*-hexane. In this study, the strong anion-exchange (SAX) sorbent **16** functioning as an XB acceptor formed an associate with perfluorinated iodoalkanes (PFIs) **17** and **18** behaving as XB donors (Figure 6) [55]. In this study, following a multidisciplinary approach, the results showed that the adsorptivities of SAX **16** for the PFIs **17** were stronger than those for the monoiodo-PFIs **18**. In accord with the negligible electrophilic properties of fluorine, SAX **16** proved to have no adsorption for hexafluorobenzene, which has no properties as an XB donor.

On the one hand, the study of Jin’s group demonstrated that the XB could also function as a pivotal noncovalent interaction in separation science. On the other hand, these results paved the way for the utilization of XB in enantioseparation science, an idea which our groups have developed successfully, starting from 2014 [56].

1. Enantioseparation of atropisomeric 4,4′-bipyridines based on XB

**Figure 7:**
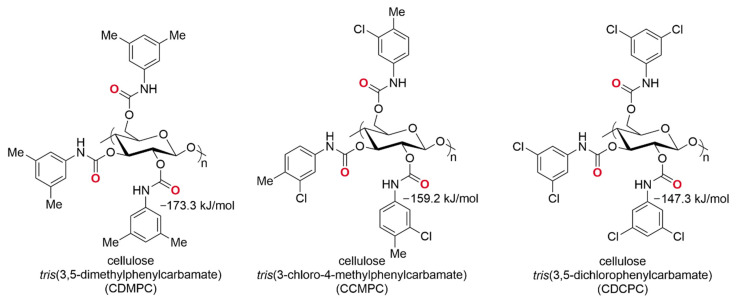
Structures of cellulose *tris*(3,5-dimethylphenylcarbamate) (CDMPC), cellulose *tris*(3-chloro-4-methylphenylcarbamate) (CCMPC) and *tris*(3,5-dichlorophenylcarbamate) (CDCPC). *V*_S,min_ values for the carbonyl oxygens (DFT/B3LYP/6-311G*) are reported.

**Figure 8:**
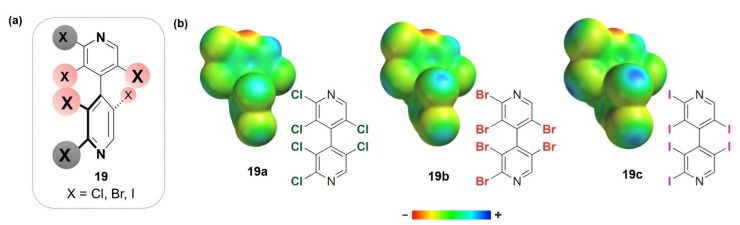
Halogenated 4,4′-bipyridines **19** (**a**), and *V*_S_ representations on electron density isosurfaces (0.002 au) for representative compounds **19a**–**c** (**b**). For the *V*_S_ representations, colors toward red depict negative *V*_S_, while colors toward blue depict positive *V*_S_, and colors in between (orange, yellow, green) depict intermediate values.

**Figure 9:**
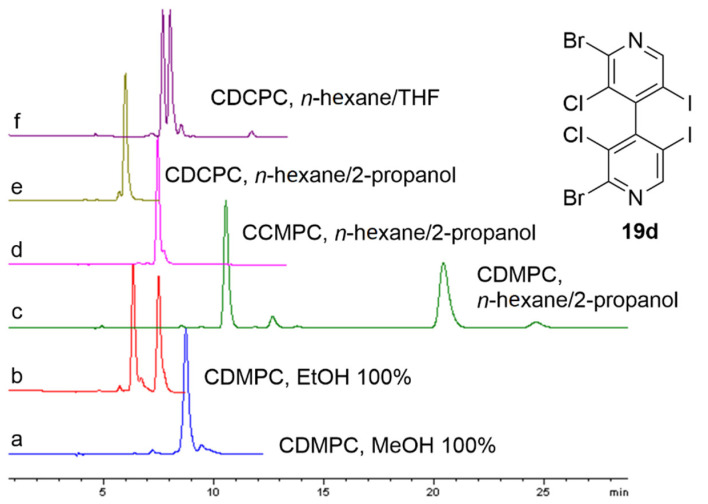
Solvent- and CSP-dependent chromatographic profiles of derivative **19d**.

By using HPLC as a technical tool, analytes as XB donors, polysaccharide-based chiral selectors as XB acceptors and using a *n*-hexane-based mixture as a mobile phase, we demonstrated that XB can drive binding and enantiorecognition of halogenated atropisomeric 4,4′-bipyridines, particularly in the case of heavy halogens, such as bromine and iodine [56,57,58]. The most important findings acquired in this field are summarized below:

(*i*) the performances of the polysaccharide carbamate-based derivatives as XB acceptors are determined by the electron charge density on the carbonyl oxygens (Figure 7). On this basis, cellulose *tris*(3,5-dimethylphenylcarbamate) (CDMPC) proved to be the chiral selector with the best XB acceptor properties because of a negative electrostatic potential minimum (*V*_S,min_) (−173.3 kJ/mol), which makes carbonyl functionality a good σ-hole acceptor, similar in terms of *V*_S,min_ and structure to other typical acceptors, such as acetone (−177.0 kJ/mol) or *N*-methylacetamide (−216.3 kJ/mol). On the contrary, chlorinated polysaccharide-based derivatives, such as cellulose *tris*(3-chloro-4-methylphenylcarbamate) (CCMPC) and *tris*(3,5-dichlorophenylcarbamate) (CDCPC), showed lower capability as XB acceptors due to the reduced electron charge density on the carbonyl oxygens (−159.2 kJ/mol and 147.3 kJ/mol, respectively);

(*ii*) as test probes functioning as XB donors with different electrophilic properties, our groups developed halogenated 4,4′-bipyridines **19** (Figure 8a) [59], where halogens serve as σ-hole sites and inductors of chirality by restricted rotation around the 4,4′-bipyridyl bond. With the aim to confirm the capability of the test probes as XB donors, some halogenated bipyridines were also studied in the solid state [58,59], and nitrogen–halogen noncovalent interactions were observed, with the percentage penetration (p.p.) of the N and halogens’ van der Waals spheres increasing from chlorine to iodine (−15.1% (N···I) ≤ p.p. ≤ −2.8% (N···Cl)). Moreover, the calculations of the electrostatic potential maxima (*V*_S,max_) allowed us to evaluate the depth of the σ-holes on chlorine, bromine and iodine, increasing in the order Cl < Br < I, as a measure of the electrophilic properties of these sites (the electron density isosurfaces of compounds **19a**–**c** are depicted in Figure 8b as representative examples); 

(*iii*) given that the enantioseparation of functionalized 4,4′-bipyridines is dependent on the substituents carried by the heteroaromatic scaffold [60], the chromatographic responses of the halogenated analogs **19** were found strictly dependent on the σ-hole depth of the halogen substituents, retention and enantioselectivity increasing from chlorine to bromine and iodine [57,58]. In this study, the use of distinct orthogonal techniques provided complementary information for a more comprehensive picture of XB-based enantiodifferentiation processes, which are the result of a balanced action of CSP, analyte and mobile phase;

(*iv*) the enantioseparation of derivatives **19** was solvent dependent and also dependent on the ability of the CSP as an XB acceptor. As shown in Figure 9 for the representative compound **19d**, the separation dropped by using polysaccharide-based selectors with lower capability as XB acceptors, namely the CCMPC and CDCPC (Figure 9d–f), compared to the CDMPC (α = 2.44, resolution (*R*_S_) = 11.3) (Figure 9c) due to the reduced XB ability of the chiral selector (*structural effect*). Moreover, the polar interaction contribution to the selectivity was progressively suppressed by changing the mobile phase from the *n*-hexane-based mixture to ethanol (α = 1.48, *R*_S_ = 2.8) (Figure 9b) and methanol (α = 1.00, *R*_S_ = 0.0) (Figure 9a) because alcohols exerted a cap effect on the carbonyls of the chiral selector by means of competitive hydrogen bonds (HBs) (*medium effect*) [57,58]; 

(*v*) in most cases, retention followed the order F < Cl < Br, which could be determined by the electrophilic σ-hole on the halogen atoms by using *n*-hexane-based mixtures, as well as by hydrophobic contacts favored in aqueous mixtures. However, it has been demonstrated that water may have little influence on the interaction energies and geometries of XB adducts in solution [61]. Thus, XB can be considered as a hydrophobic equivalent of the hydrophilic hydrogen bond [62], potentially also acting in water-containing mobile phases. Coherently, halogen-dependent enantioseparations were also obtained by using acetonitrile/water mixtures as mobile phases [58]; 

(*vi*) molecular dynamics (MD) simulations were performed by using a nonamer framework of the CDMPC polymer, 16 bipyridine enantiomers, *n*-hexane as explicit solvent and the explicit σ-hole approach [63] to properly account for the anisotropic distribution of the electron density on halogens [58,64]. This computational approach confirmed the presence of halogen–oxygen contacts governing the dynamics of the enantioseparations, with interesting geometric and penetration parameters. Moreover, in this study, the involvement of different sites was statistically evaluated over 10 nanoseconds of MD. As a result, for the iodinated derivative, the carbonyls of the virtual chiral selector were shown to be the most frequent recognition sites, whereas for the chlorinated system, a wider distribution was observed without any specific involvement of the carbonyl sites [64].

Later, studies integrating experimental analysis and computational techniques, such as *V* analysis and MD simulations, demonstrated that XB, as a selector–selectand intermolecular interaction, is less effective in the presence of strong HBs or π–π interactions in the enantioseparation of 4,4′-bipyridine derivatives with amylose-based selectors, whereas it has shown effective tuning ability with cellulose-based selectors, even in the presence of HBs contributing to enantiodifferentiation [65].

2. Enantioseparation of planar chiral ferrocenes based on XB

**Figure 10:**
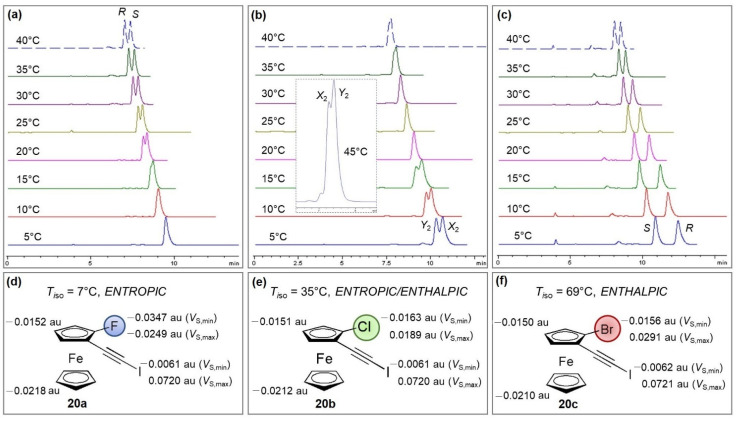
Enantioseparation of ferrocenes **20a** (**a**), **20b** (**b**) and **20c** (**c**) at variable temperatures on a CDMPC-based chiral column with *n*-hexane/2-PrOH 95:5 *v*/*v* as mobile phase and variation of the *V*_S,min_ and *V*_S,max_ values (au, atomic unit) (**d**–**f**) as the 1-halogen substituent changes in the series of 1-halo-2-(iodoethynyl)ferrocenes **20a**–**c** (Reprinted/adapted with permission from Ref. [67]. Copyright 2022, Elsevier).

Very recently, the impact of halogen type and position on the enantioseparation of halogenated ferrocenes [66] was investigated with polysaccharide-based chiral selectors by correlating theoretical and experimental data [67]. For this purpose, thermodynamic quantities associated with the enantioseparations were derived from van’t Hoff plots, and for 1-halo-2-(iodoethynyl)ferrocenes (1-halogen = F, Cl, Br) **20**, halogen-dependent thermodynamic profiles were identified on a CDMPC-based column (Figure 10). In particular, with the aim to unravel the functions of halogen substituents in mechanisms underlying the selector–selectand complex formation at the molecular level, local electron charge densities of specific molecular regions of the interacting partners were evaluated in terms of calculated *V* and related source function contributions [68]. On this basis, it was demonstrated that HB involving the NH group of the selector, as the HB donor, participates in the enantiodifferentiation mechanism for the fluorinated ferrocene **20a**, whereas an XB involving the carbonyl group of the selector occurred for the more polarizable 1-bromoferrocene **20c** [67].

3. Miscellaneous

After the pioneering investigations on XB-based enantioseparations discussed above, a number of other studies hypothesized the participation of XBs in enantioseparation processes on polysaccharide-based chiral selectors [69,70,71,72,73,74]. In this regard, some open questions emerge:

(*i*) the involvement of fluorine in XB in enantioseparation science is rather unlikely [75]. In fact, fluorine is a very weak XB donor due to its high electronegativity and low polarizability, and it was shown to act as an electrophile only when bound to strong electron-withdrawing groups, such as CN or F [6];

(*ii*) very recently, XB has been proposed as a noncovalent interaction involved in the enantioseparation of some chlorinated compounds on polysaccharide-based CSPs [69,72,73]. In this regard, a theoretical re-examination of these hypotheses evidenced that the electronic properties of chlorine as a substituent make this atom a weak XB donor; thus, a poor involvement in XB is expected for this halogen, particularly in competitive systems, where HB can also participate in enantiorecognition [48,64];

(*iii*) in other studies, it was hypothesized that in chlorinated polysaccharide-based selector, the chlorine substituent may participate in XB with analytes functioning as XB acceptors [70,71,74]. Recently, theoretical calculations demonstrated the presence of electrophilic σ-holes on chlorines but small in magnitude [48]. On this basis, the potential of these chlorines to be involved in XBs is rather limited, particularly considering that the corresponding N-H moieties of the chlorinated selectors are stronger competitive electrophilic sites. Thus, it is likely that analytes with properties like XB and HB acceptors show a preference toward N-H. On the other hand, in the 1980s, Okamoto et al. assumed that in cellulose phenylcarbamates, polar substituents located on the phenyl rings of the phenyl carbamate moiety participated in non-enantioselective noncovalent interactions with chiral analytes because they are located far from a chiral glucopyranose unit [76], without any favorable contribution to enhancing enantioselectivity. On this basis, it is likely that the chlorine of the polysaccharide-based selectors has no other function beyond that of modulating the electron charge density on the carbamate sites.

#### 3.1.4. Supercritical Fluid Chromatography (SFC) Enantioseparations

The effect of non-polar pressurized carbon dioxide on XB-driven enantioseparations was also explored under SFC conditions [48]. Carbon dioxide is considered a hexane-like solvent with respect to its elution strength [77]. Thus, changing *n*-hexane to carbon dioxide should cause no relevant changes in retention and selectivity [78]. Actually, carbon dioxide is not really hexane-like because it is more polarizable, and it has local dipoles (C=O bonds) and partial charges on both carbon and oxygen [78]. Moreover, theoretical calculations and related experimental data proved that carbon dioxide can participate in XBs (O=C=O···X) acting as XB acceptor [79]. In this perspective, the results of a series of comparative enantioseparations performed under SFC (carbon dioxide/2-propanol 9:1) and NPLC (*n*-hexane/2-propanol 9:1) conditions on CDMPC demonstrated a strong impact of carbon dioxide on the enantioseparation of halogenated derivatives containing strong XB donor sites [48].

#### 3.1.5. Self-Disproportionation of Enantiomers (SDE)

**Figure 11:**
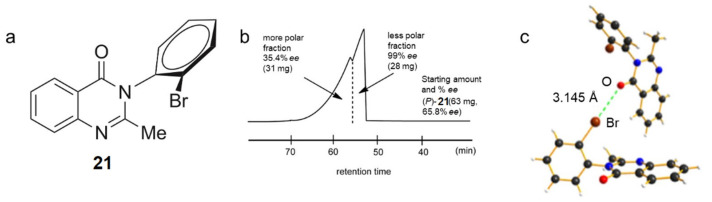
(**a**) Structure of mebroqualone (**21**); (**b**) MPLC chart of **21**; (**c**) XB in the crystal packing of *rac*-**21** (Reprinted/adapted with permission from Ref. [80]. Copyright 2017, Wiley).

Soloshonok et al. reported the first example of XB-driven self-disproportionation of enantiomers (SDE) performed through achiral medium-pressure liquid chromatography (MPLC) and gravity-driven chromatography [80]. This phenomenon is based on the self-association of enantiomers, yielding fractions of nonracemic samples, which are enriched and/or depleted in one of the constituent enantiomers under achiral chromatographic conditions [81]. In MPLC experiments using *n*-hexane-containing mixtures as mobile phases and mebroqualone (**21**) as analyte (Figure 11a), two clear peaks with a distinct boundary between them were observed (Figure 11b). By using a preparative MPLC column and starting from (*P*)-**21** (>65.8% enantiomeric excess (*ee*)), the authors separated the two peaks, and their enantiomeric composition was analyzed by enantioselective HPLC.

The data obtained revealed that the less polar fraction contained enantiomerically pure (*P*)-**14** (>99% *ee*). Otherwise, the more polar fraction provided enantiomerically depleted (*P*)-**14** (35.4% *ee*). The results of a crystallographic study suggested the possibility of an XB between C=O groups and the *ortho*-bromine atoms in the racemic crystals, the observed distance C=O···Br being 3.145 Å (Figure 11c). The racemic crystals proved to be more stable than those of (*P*)-**14**, where XBs were not observed. If such interactions are still present in solution, it is reasonable to argue that, under chromatographic conditions, the first fractions are monomers of the excess enantiomers, and the last fractions correspond to heterochiral XB high-order species representing the racemic portion of the considered sample.

### 3.2. Enantioseparations Based on ChB

On the basis of the most recent findings, XB appears to be a new code by which chiral molecules can relate to each other in enantioseparation science. On the other hand, other interactions involving electrophilic σ-holes, such as ChB, could be envisaged to perform the same function. Enantioseparations of selenium and sulfur derivatives were reported in the literature, and higher retention and selectivity were observed for selenium compounds compared to sulfur analogs [82,83,84]. Nevertheless, in general, the impact of σ-holes of sulfur and selenium in enantioseparation science has been neglected, and until recently, ChB remained explored to a lesser degree in this field.

**Figure 12:**
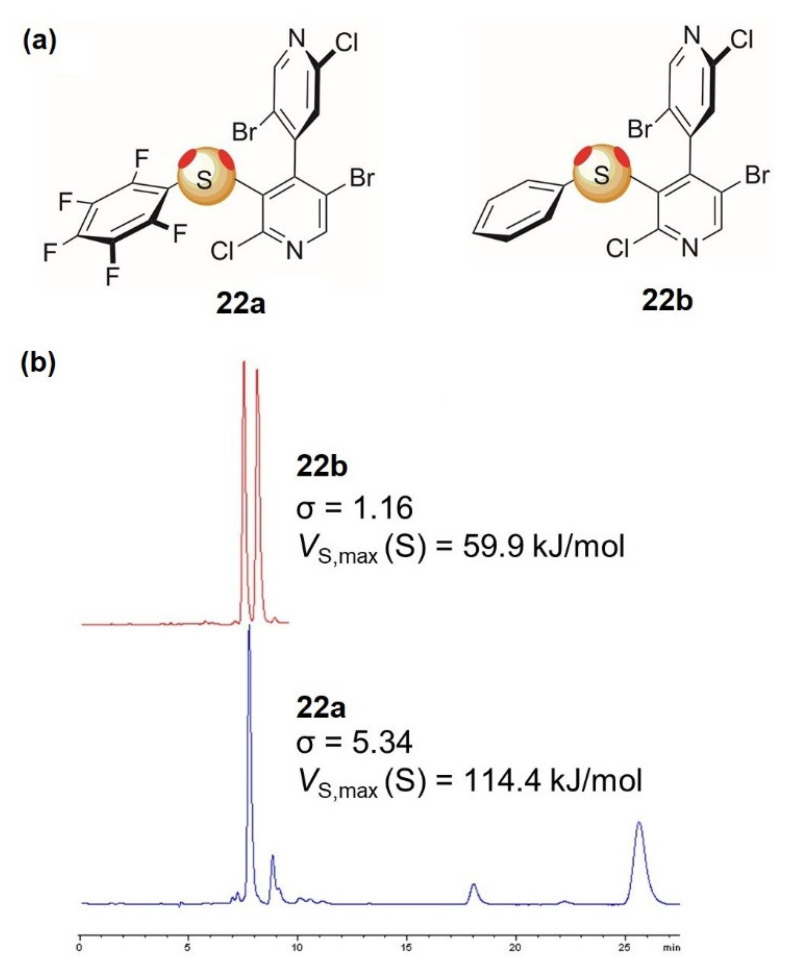
(**a**) Structure of derivatives **22a** and **22b**; (**b**) comparative enantioseparation of compounds **22a** and **22b** (CDMPC, *n*-hexane/2-propanol) (Reprinted/adapted with permission from Ref. [80]. Copyright 2018, Elsevier).

Following the studies on XB-based enantioseparations, later, we also considered ChB, and, again exploiting the 4,4′-bipyridine core, we designed and prepared bipyridines **22** containing sulfur or selenium as electrophilic chalcogen sites [85,86,87,88]. As mentioned above, in this case, due to the valence of the atoms, two σ-holes could be identified. Thus, in the new 4,4′-bipyridine motif, the σ-holes on sulfur and selenium were electronically differentiated by a proper substitution. The design was computationally guided by using the *V* analysis and the source function electrostatic potential decomposition applied to the sulfur- and selenium-centered σ-holes [86,87,88]. On this basis, the pentafluorophenyl group (**22a**) proved to be effective for the purpose of enhancing the electrophilic ability of one σ-hole, whereas the second one was blocked using the atropisomeric structure as a steric tether (Figure 12a). As a result, high selectivity was observed for the fluorinated derivative **22a**, confirming the pivotal role of the pentafluorophenyltio group as the “enantioseparation driver”, while poor selectivity was observed for the nonfluorinated compound **22b** (Figure 12b) [86].

Further to these results, we observed that the pentafluoro derivative presented an additional region of electronic charge depletion centered on the fluorinated aromatic ring, which could be involved in the enantiodiscrimination process forming the so-called π-hole bond, conceptually analogous to the σ-hole bond and involving an unpopulated π* orbital, the π-hole, as a donor. With the aim of gaining insights on the function of these holes, for a large series of compounds containing fluorinated rings and electrophilic sulfur and selenium centers, the values of the second eluted enantiomers were correlated to the *V*_S,max_ values associated with electrophilic regions. Interesting correlations were observed, which enable us to reasonably conclude that both ChBs and π-hole bonds can function as noncovalent interactions in enantioseparation science.

## 4. Enantioselective Complexation

Enantioselective complexation is the process by which a host molecule selectively recognizes and binds one chiral guest over its enantiomer [89]. σ-hole interactions were also exploited in this field for the enantioselective recognition of anionic or neutral substrates [25,29].

### 4.1. Recognition of Anionic Guests

**Figure 13:**
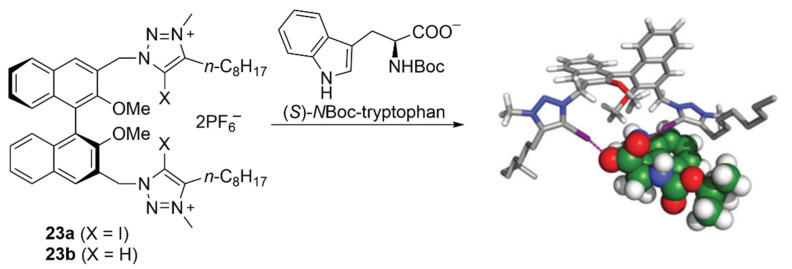
Effect of XB on the enantioselective discrimination of chiral anions (Reprinted/adapted with permission from Ref. [90]. Copyright 2016, Royal Chemical Society).

In 2016, by using chiral receptor **23a**, Beer et al. observed for the first time the positive influence of XB on the enantioselective discrimination of different amino-acid-derived chiral oxoanions. Indeed, these anions interacted with receptor **23a** through highly directional, almost linear I···O XBs, whereas angles in the range 130–140° were observed for HBs with the non-iodinated receptor **23b**. The sterically bulky indole moiety of *N*Boc-tryptophan resulted in the largest selectivity of 1.67 with preference for the (*S*)-enantiomer with **23a**, whereas **23b** led to very low stereo-induction (Figure 13) [90]. A quite similar selectivity of 1.69 was obtained by employing the neutral halogenated tetradendate receptor [91].

**Figure 14:**
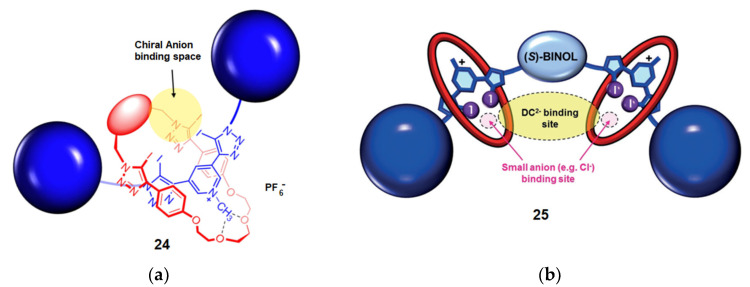
Design of XB-based [2]-rotaxane **24** (**a**) and [3]-rotaxane **25** (**b**) for chiral recognition of anions (Reprinted/adapted with permission from Ref [92]. Copyright 2017, American Chemical Society and from Ref [93]. Copyright 2018, Wiley).

The role of XB in the chiral discrimination of anions was again demonstrated by Beer et al. by using [2] and [3]rotaxanes [92,93] (Figure 14). It was shown that [2]rotaxane **24** was more effective than the individual non-interlocked components, giving a selectivity of up to 2.93 for the (*S*)-*N*Boc-proline anion. Molecular dynamics (MD) simulations revealed that the linear XBs between the anion and the rotaxane cavity allowed the guest to be in close proximity of the chiral (*S*)-BINOL unit [92] (Figure 14a). Based on the same principle, a more sophisticated [3]rotaxane **25**, bearing two binding sites around a chiral (*S*)-BINOL unit, was developed for the recognition of dicarboxylate anions. A good discrimination of chiral *N*Boc-glutamate was observed with a selectivity of 5.7 for the (*S*)-glutamate enantiomer [93] (Figure 14b).

**Figure 15:**
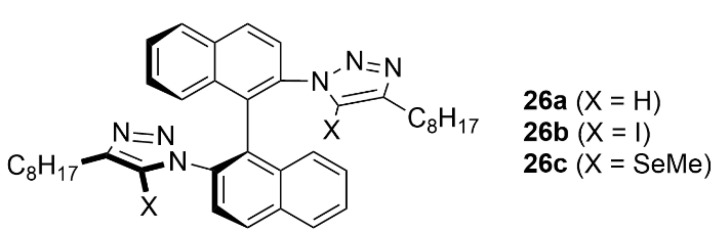
Structure of chiral hosts **26a**–**c**.

Finally, with the aim to compare the influence of XB, ChB and HB on the chiral selectivity and sensing of dicarboxylate anions, three structurally related chiral host systems **26a**–**c** bearing I, Se and H were prepared. The XB-based host **26b** performed the best in the chiral recognition of chiral dicarboxylates (glutamate and tartrate) and, together with the ChB-based derivative **26c**, allowed higher discrimination of dicarboxylate geometric isomers (fumarate/maleate and phthalate/isophtalate). Moreover, the ChB-based host displayed diagnostic fluorescence responses to different dicarboxylate geometric isomers and enhanced stability toward highly basic oxoanions in aqueous solvent media (Figure 15) [94].

### 4.2. Recognition of Neutral Guests

**Figure 16:**
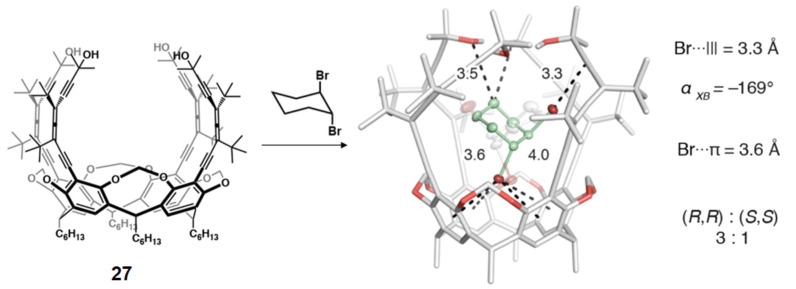
Chiral recognition of *trans*-1,2-dibromocyclohexane in the diaxial conformation bound to the interior of **27** (Reprinted/adapted with permission from Ref [95]. Copyright 2017, American Chemical Society).

The enantioselective complexation of neutral molecules emphasizing the involvement of XB was also explored in 2017 by two different research groups. Diederich et al. studied the chiral recognition properties of enantiopure alleno-acetylenic cage receptor **27** toward (±)-*trans*-1,2-dihalocyclohexanes and (±)-*trans*-1,2-dimethylcyclohexane [95]. While the latter formed a weak complex with **27** (*K*_a_ = 110 M^−1^), much stronger complexes were obtained with (±)-*trans*-1,2-dichloro- and (±)-*trans*-1,2-dibromocyclohexane (*K*_a_ = 3800 M^−1^ and 29,000 M^−1^, respectively). The higher values for the halo derivatives were the result of strong directional XBs between halogens and acetylene/aryl groups, as observed in the co-crystal structures. The best chiral recognition result was observed for the 1,2-dibromo derivative with a ratio of 3:1 for the diastereomeric complexes formed by the (*R*,*R*)- and (*S*,*S*)-enantiomers with **27** (Figure 16).

**Figure 17:**
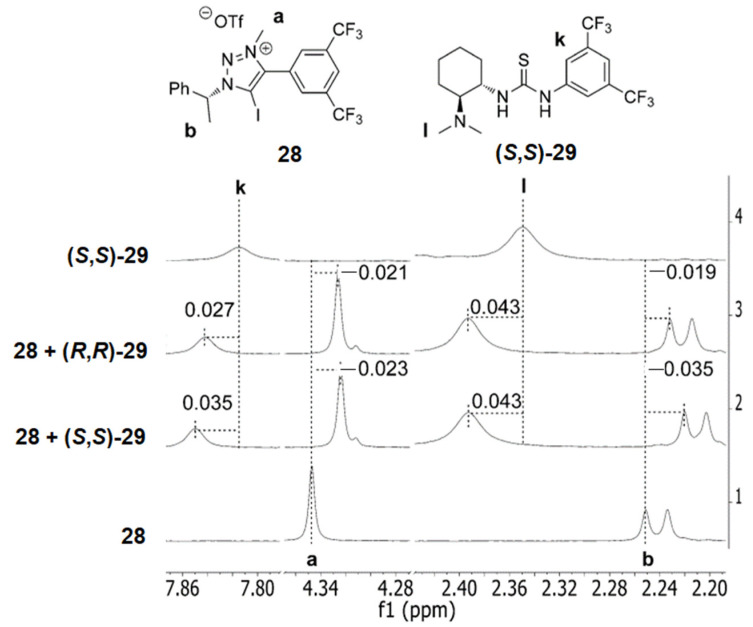
Structures of **28** and **29** and recorded ^1^H NMR shifts in CDCl_3_ for their 1:1 mixtures (Reprinted/adapted with permission from Ref [96]. Copyright 2017, Wiley).

Kanger et al. reported a chiral XB donor **28** able to interact with urea **29** and behaving differently in the presence of either its (*S*,*S*) or (*R*,*R*) enantiomer, as shown by ^1^H NMR [96]. Indeed, the different NMR shifts compared to the free XB donor obtained with (*S*,*S*)-**29** (+0.035 ppm) and (*R*,*R*)-**29** (+0.027 ppm) confirmed the enantiodiscrimination ability of **28** (Figure 17). XB probably occurred between the I and S atoms, with possible HB involving of the NH of **29** and the negative belt of iodine in **28**.

## 5. Asymmetric Catalysis

### 5.1. XB-Based Catalyzed Asymmetric Reactions

**Figure 18:**
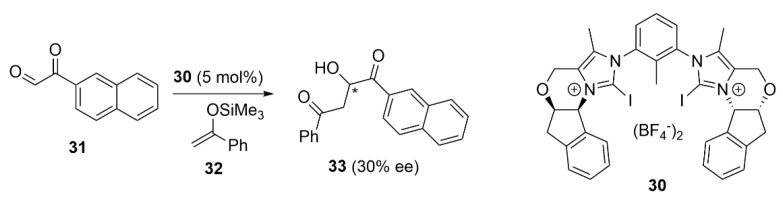
XB-Catalyzed asymmetric Mukaiyama reaction.

The enantiodiscrimination property of XB donors suggests that potential enantioselectivity induction in asymmetric reactions could be expected. In this regard, Huber et al. developed the bidentate XB donor **30** and demonstrated its ability of enantiodiscrimination toward several diamines and diols through XB formation [97]. More interestingly, **30** was used as a chiral organocatalyst in the Mukaiyama reaction of ethyl glyoxylate **31** with TMS-enolate **32**, furnishing the aldol product **33** with 30% ee. This report represents the first example of enantioselective reaction involving only the σ-hole interactions (Figure 18).

**Figure 19:**
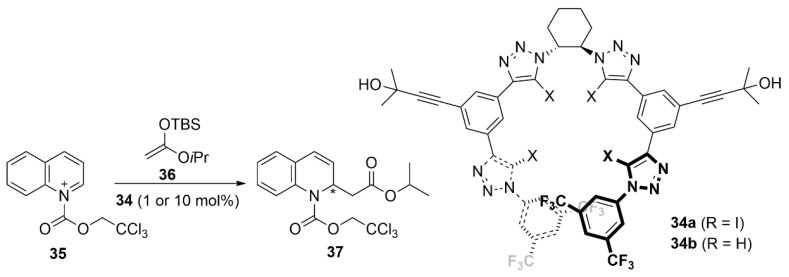
XB-catalyzed asymmetric Reisser-type reaction.

Similarly, García Mancheño et al. described the tetradentate iodotriazole **34a** and its non-iodinated analog **34b** as enantiopure receptors for chiral mono and dicarboxylate anions [98]. In particular, titration of XB donor **34a** with the tartrate salts revealed a good selectivity of 2.10 for the D-enantiomer of the tartrate tetrabutylammonium salt. The two receptors were then used as catalysts in the Reissert-type dearomatization of activated quinoline **35** with silylketeneacetal **36**. Interestingly, while the (*S*)-product **37** was favored with the **34a** catalyst, (*R*)-**37** was formed preferentially with the non-iodinated catalyst **34b**. Moreover, whereas at high catalyst loading (10% cat), a higher ee was obtained with **34b** (58% *ee* vs. 30% *ee*), **34a** performed better at lower loading (1% cat: 28% *ee* vs. 18% *ee*). These results suggest that catalyst **34a** is more able to outcompete the uncatalyzed background reaction, which produces 41% of the racemic product under the same conditions. CD experiments showing opposite Cotton effects for the two catalysts suggest a different spatial organization. Additional DFT calculations showed that the introduction of the large iodine atoms as substituents to the **34b** scaffold caused a distortion of the helical cavity that prevented a higher coordination number. These results explain the observed reversed selectivity between the two catalysts (Figure 19).

**Figure 20:**
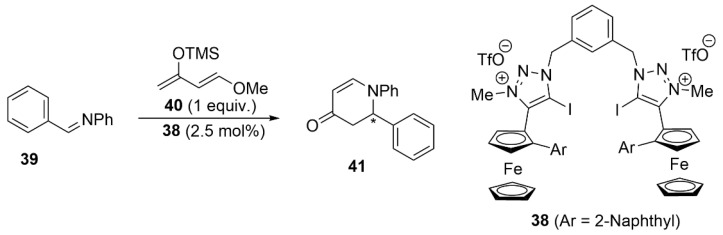
XB-catalyzed asymmetric aza-Diels–Alder reaction.

More recently, our groups described the bis-iodotriazolium catalyst **38** bearing planar chirality of ferrocene. This bidendate catalyst performed efficiently in the aza-Diels–Alder reaction between imine **39** and Danishefsky diene **40**, but product **41** was obtained with a very low *ee* of 6%, probably because of the high flexibility of the two iodotriazolium arms of the catalyst [99] (Figure 20).

**Figure 21:**
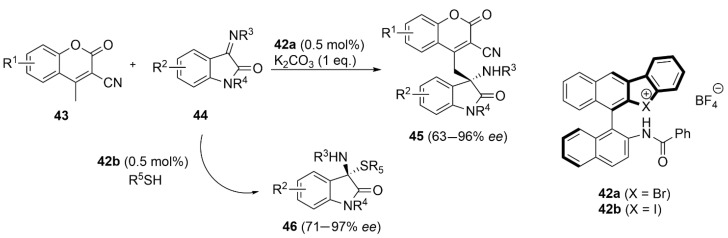
XB/HB-catalyzed asymmetric additions to ketimines **44**.

Bifunctional chiral catalysts able to conduct XBs and HBs were found to be much more efficient in terms of enantioselectivity induction than the previous catalysts based only on XBs. In recent years, very limited chiral backbones were used by different authors. Yoshida et al. developed chiral binaphthyl-based halonium derivatives **42** bearing an amino functionality. The bromonium catalyst **42a** was used in the vinylogous Mannich reaction of cyanomethyl coumarins **43** with ketimines **44**, delivering the coupling products **45** in high yields and *ee* up to 96% [100]. The iodonium catalyst **42b** was, however, more powerful in the asymmetric addition of bulky thiols to ketimines **44** for the formation of N,S-acetal products **46** in high yields and enantioselectivities [101]. In both cases, the DFT-calculated plausible key intermediate structures showed that both XBs and HBs concur with the observed high enantioselectivity induction (Figure 21).

**Figure 22:**
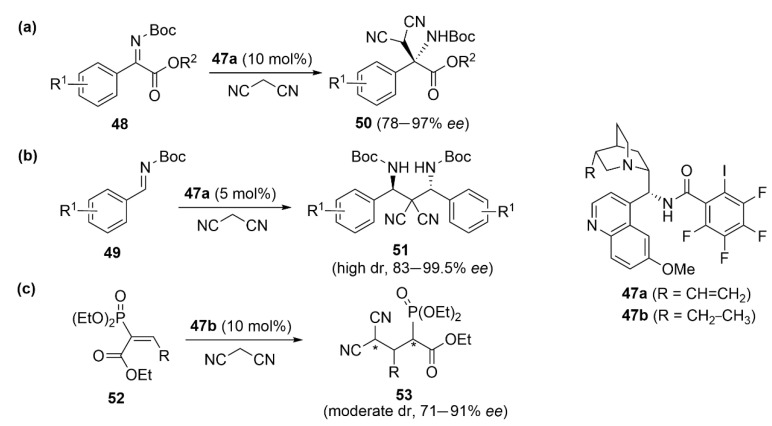
XB/HB-catalyzed asymmetric reactions with *Cinchona* alkaloid-derived chiral catalysts **47** involving iminoesters (**a**), imines (**b**) and vinyl phosphonates (**c**).

Arai et al. employed the *Cinchona* alkaloid-derived chiral catalysts **47a** bearing iodine installed on a pentafluorobenzene ring as an XB donor, an amidic NH group as an HB donor and an amine as a basic site. This catalyst was found to be very efficient in the asymmetric Mannich-type reaction of malononitrile with *N*-Boc α-ketiminoesters **48** [102] and in the enantio- and diastereoselective double Mannich reaction of malononitrile with *N*-Boc imines **49** [103]. Products **50** and **51** were obtained in high yields and *ee*, and in both cases, the role of XB was confirmed by using model compounds lacking iodine functionality (Figure 22a,b). A very similar chiral catalyst **47b** was used by Kanger et al. in the highly enantioselective Michael addition of malononitrile to vinyl phosphonates **52** to generate coupling product **53** [104] (Figure 22c). The authors demonstrated an interesting effect of the NH group, which enhanced the Lewis acidity of iodine by establishing an intramolecular I···H HB. In a very recent publication, the same authors employed a simplified chiral backbone possessing XB, HB and Lewis base properties [105]. This multi-functional catalyst based on the chiral *trans*-cyclohexane diamine backbone was applied in the enantioselective Mannich reaction between malononitrile and diphenylphosphinoyl-protected aldimine, affording products in high yields (74–98%) and moderate to high *ee* (70–89%). However, the control experiments with model compounds bearing other halogens instead of iodine showed that the enantioselectivity is controlled by HB and not by XB.

### 5.2. PnB-Based Catalyzed Asymmetric Reactions

**Figure 23:**
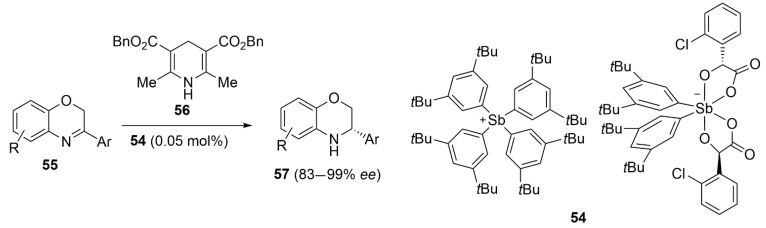
PnB-catalyzed asymmetric reduction in benzoxazines **55**.

Tan et al. reported the first case of asymmetric PnB catalysis by using the chiral antimony(V) cation/anion pair **54** [106]. The high performance of this catalyst was proven in enantioselective transfer hydrogenation reaction with a low catalyst loading. Indeed, the reduction in benzoxazines **55** with Hantzsch ester **56** performed very well with only 0.05 mol% of catalyst, and *ee* as high as 98% were obtained for products **57**. The control experiments revealed that the high catalytic activity of **54** is based on PnB of Sb^+^ combined with the Lewis basic property Sb^−^ (Figure 23).

### 5.3. ChB-Based Catalyzed Asymmetric Reactions

**Figure 24:**
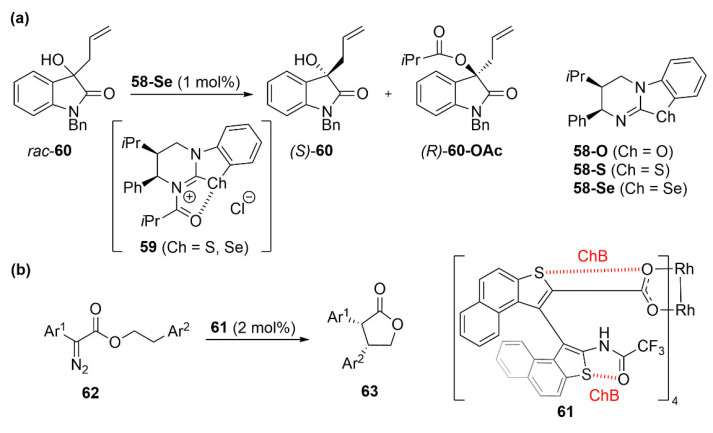
Stabilization of intermediate or catalyst through ChB in asymmetric reactions: (**a**) kinetic resolution of tertiary alcohols and (**b**) intramolecular C−H insertion into α-aryl-α-diazoacetates.

Although efficient ChB-based asymmetric catalysis relying on the direct interaction of the catalyst with the substrate is still unreported, the importance of ChB for the stabilization of intermediates or catalysts was recently described in two asymmetric reactions. By studying a series of isochalcogenourea catalysts **58** (X = O, S, Se), Smith et al. demonstrated that the higher activity and selectivity of the sulfur- and selenium-based catalysts in different catalytic reactions was due to the increased stability of the *N*-acetylated intermediates **59** allowed by strong intramolecular S···O and Se···O ChBs. For instance, **58**-Se at 500 ppm catalyst loading allowed the kinetic resolution of tertiary alcohols **60** with conversions in the range of 31–50% and selectivity between 70 and 120 (Figure 24a) [107]. A very recent example by the same group highlighted again the importance of S···O ChB in asymmetric organocatalysis [108].

Finally, Furuta et al. showed that conformational control of the dirhodium catalyst **61** by ChB led to an increased enantioselectivity in the catalytic intramolecular C−H insertion into α-aryl-α-diazoacetates **62** for the formation of cis-α,β-diaryl γ-lactones **63** [109]. Indeed, X-ray diffraction analysis indicated that the Rh centers in this catalyst are embedded in a defined chiral environment arising from the presence of intramolecular ChBs (Figure 24b).

## 6. Conclusions

After the definition of the σ-hole concept at the beginning of the twenty-first century, the ability of many atoms to drive noncovalent interactions could be rationalized, offering new opportunities in chemistry, as shown by the increasing number of applications. In the present review, we focused on the applications where the σ-hole interaction was demonstrated to play a key role in the control over chirality. To date, the emerging applications are limited to the formation of chiral supramolecular assemblies, separation of enantiomers, enantioselective complexation and asymmetric catalysis.

However, this field is still in its infancy, and many improvements and new developments are expected in future years. While most current applications are based on XB, the emergence of new σ-hole interactions, such as ChB and PnB, should widen their utility in stereoselective processes.

## Figures and Tables

**Table 1:**
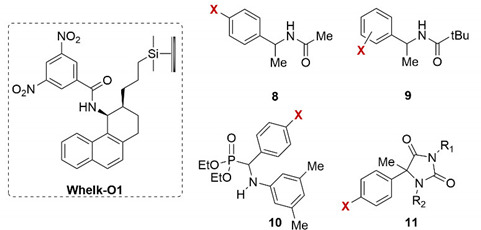
Halogen effect on the enantioseparation of derivatives **8**–**11** on Whelk-O1 [50,51].

Compound	X	R_1_	R_2_	*k*_1_^1^	α ^2^
**8**	H	-	-	3.72	3.17
**8**	*p*-I	-	-	4.10	5.12
**9**	H	-	-	1.39	6.74
**9**	*p*-F	-	-	1.17	7.29
**9**	*p*-Cl	-	-	1.48	11.6
**9**	*p*-Br	-	-	1.61	12.8
**9**	*p*-I	-	-	1.75	13.7
**9**	*m*-Br	-	-	1.61	13.1
**10**	H	-	-	0.87	1.29
**10**	*p*-F	-	-	0.83	1.39
**10**	*p*-Cl	-	-	0.84	1.55
**10**	*p*-Br	-	-	0.86	1.66
**11**	F	H	H	0.62	2.06
**11**	F	Me	H	1.17	2.52
**11**	F	Me	Me	3.59	3.40
**11**	Cl	H	H	0.62	2.61
**11**	Cl	Me	H	1.22	3.31
**11**	Cl	Me	Me	3.81	4.96
**11**	Br	H	H	0.66	2.82
**11**	Br	Me	H	1.31	3.58
**11**	Br	Me	Me	3.97	5.20
**11**	I	H	H	0.69	2.97
**11**	I	Me	H	1.41	3.90
**11**	I	Me	Me	4.29	5.80

^1^ Retention factor of the first eluted enantiomer. ^2^ Selectivity factor.

## Data Availability

Not applicable.
